# Children’s active commuting to school: an interplay of self-efficacy, social economic disadvantage, and environmental characteristics

**DOI:** 10.1186/s12966-015-0190-8

**Published:** 2015-02-28

**Authors:** Wenhua Lu, E Lisako J McKyer, Chanam Lee, Marcia G Ory, Patricia Goodson, Suojin Wang

**Affiliations:** Silver School of Social Work, New York University, 20 Cooper Square, Room 240, New York, 10003 USA; Department of Health & Kinesiology, Texas A&M University, College Station, TX 77840-4243 USA; Department of Landscape Architecture and Urban Planning, College of Architecture, Texas A&M University, College Station, TX 77843-3137 USA; Health Promotion & Community Health Sciences, Health Science Center, School of Public Health, Texas A&M University, 1266 TAMU, College Station, TX 77843-1266 USA; Department of Statistics, Texas A&M University, 3143 TAMU, College Station, TX 77843-3143 USA

**Keywords:** Active commuting to school, Self-efficacy, Social economic disadvantage, Environment

## Abstract

**Background:**

Active commuting to school (ACS) can promote children’s physical activity and may help prevent childhood obesity. Previous researchers in various disciplines, e.g., health, urban planning, and transportation, have identified various predictors of ACS. However, little research has been carried out into investigating the effect of self-efficacy on ACS. The purpose of this study is to investigate the roles of children’s and parents’ self-efficacy in children’s ACS, controlling for sociodemographic and objective environmental characteristics.

**Methods:**

This study is part of the Texas Childhood Obesity Prevention Policy Evaluation (T-COPPE) project, which includes data from 857 parent/child pairs from 74 schools who lived within two miles of school in Texas. Measures included children’s usual modes of commuting to school, participants’ sociodemographics, perceived self-efficacy toward ACS, sources of children’s self-efficacy, school settings, and objective environmental constraints. Multilevel structural equation modeling (SEM) was employed to test the hypothesized pathways using M*plus* 7.0.

**Results:**

Around 18% of the children were active commuters. Two sources of children’s self-efficacy were identified, i.e., emotional states (β = 0.36, p < 0.001) and social modeling (β = 0.28, p < 0.01). Compared with children’s self-efficacy (β = 0.16, p < 0.001), parents’ self-efficacy (β = 0.63, p < 0.001) had a stronger influence on children’s ACS. Participants’ social economic disadvantage (β = 0.40, p < 0.001), environmental constraints (β = −0.49, p < 0.001), and school setting (β = −0.17, p = 0.029) all had statistically significant direct effects on children’s ACS.

**Conclusions:**

Future initiatives should consider both parents’ and children’s self-efficacy in developing strategies for promoting children’s ACS. Social disadvantage and environmental constraints also need to be addressed for effective interventions. The work reported here provides support for the continuing exploration of the role of self-efficacy in children’s ACS.

## Background

Recently, the National Poll on Children’s Health recognized childhood obesity as the leading health concern among parents in the U.S., topping drug abuse and smoking [1]. The prevalence of obesity nearly tripled among American children and adolescents in the past 30 years, which has brought along various health problems that were not seen until adulthood, including high blood pressure, type 2 diabetes, and elevated blood cholesterol levels [2,3]. Considering the health consequences of childhood obesity and that more children are becoming overweight, preventing and reducing childhood obesity is an important public health challenge.

Recent research has acknowledged the role of active commuting to school (ACS), for example, walking or biking to/from school, in promoting children’s physical activity and its potential for preventing and reducing childhood obesity [4,5]. For example, Mendoza et al. conducted a cluster randomized controlled trial of the Walking School Bus program in Texas and reported significant increases of daily moderate-to-vigorous physical activity to the intervention students compared with the control students [5]. Despite the health benefits of ACS, the percentage of children who walk or bike to school has declined dramatically in the U.S. over the past few decades, from 47.7% in 1969 to 12.7% in 2009 [6]. It is critical that effective interventions be developed and conducted to reverse the declining trend.

Over the past decades, researchers in various disciplines, e.g., health, urban planning, and transportation, have identified multiple personal, environmental, and social factors associated with ACS [7,8]. However, little research has been carried out into investigating psychological factors that may influence children’s ACS [7]. Examination of psychological factors within the ACS context is critical to understanding and implementing effective interventions, because 1) most interventions that placed emphasis on structural or environmental improvements have proved insufficient in changing children’s commuting behavior to school and 2) research has established the predictive power of multiple psychological factors on promoting children’s physical activity, including attitudes, perceived barriers, and self-efficacy [9-11].

Self-efficacy is one of the strongest and most widely acknowledged determinants of health behavior in general [12]. Among children and adolescents, self-efficacy has also been identified as a consistent variable associated with physical activity [11]. For example, a Californian study conducted among 213 six-grade students substantiated that among both boys and girls, physical activity self-efficacy was the strongest independent predictor of daily participation in vigorous physical activity [13]. As a social cognitive construct, self-efficacy refers to individuals’ self-beliefs in their ability to control their functioning, overcome difficulties, and perform specific tasks [12]. Previous ACS studies have also confirmed the important role of parental self-efficacy in children’s active commuting behaviors, showing that higher parental self-efficacy was positively associated with children’s ACS [5,14]. However, it is unclear whether and how children’s self-efficacy can influence their *own* behavior of ACS. Children, like adults, are able to contribute meaningful research data; their belief of their own abilities to navigate physical and social environments that they may encounter when actively commuting to school need to be recognized and investigated.

Further, previous ACS studies focused mainly on parents, based on the hypothesis that parents play a greater role than children in choosing the mode of travel to school [15]. However, there’s no empirical evidence supporting this hypothesis. A comparison of parents’ versus children’s self-efficacy in predicting children’s ACS may provide supporting or opposing evidence for this hypothesis.

Therefore, the purpose of this study was to investigate the roles of both children’s and parents’ self-efficacy in children’s ACS based on Bandura’s social cognitive theory (SCT). Specifically, we aimed to 1) determine the association between children’s self-efficacy and their ACS behavior, 2) explore the sources of children’s self-efficacy, 3) compare the power of children’s vs. parents’ self-efficacy on predicting/explaining children’s ACS, and 4) examine the relationship between children’s and parents’ self-efficacy.

### Theoretical framework

According to Bandura’s SCT, individuals’ behavior is determined by the interaction among personal, behavioral, and environmental factors [16]. Further, individuals’ beliefs of their capabilities affect their decisions about whether a behavior will be adopted and maintained [12]. In the context of ACS, children’s self-efficacy for *scheduling* regular ACS, *seeking social support* for ACS, and overcoming different kinds of *barriers* to ACS may influence their active commuting behavior [12,17].

Baudura also hypothesized that people’s self-efficacy can be developed by different sources of influence, including mastery experience, vicarious experience or social modeling, verbal persuasion, and emotional and physiological states [12]. When applied to ACS, children may be more likely to adopt active transport if they have asked their parents for permission to ACS (*previous experience*), if they observed that people around them walked or biked often (*vicarious experience/social modeling*), if their parents or schools have persuaded them to walk or bike (*verbal/social persuasion*), or if they feel safe or happy walking or biking to school (*emotional/physiological states*).

**Figure 1:**
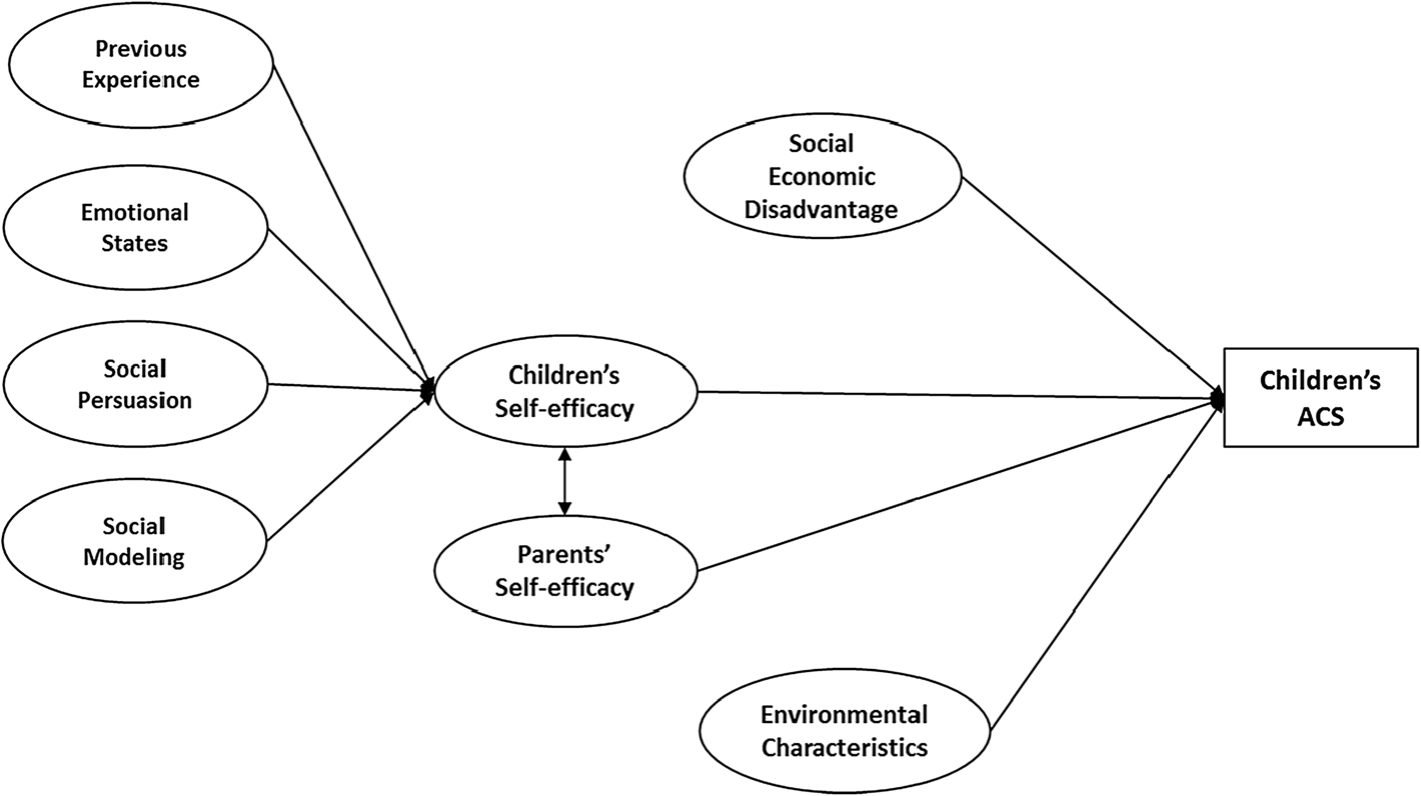
**Theoretical framework.**

For this study, we developed a theoretical framework based on the SCT. As presented in Figure 1, we hypothesized that controlling for participants’ sociodemographics and environmental constraints, children’s self-efficacy is positively associated with their ACS (Hypothesis #1); children’s previous experience of asking for permission to ACS, emotional states, the persuasive messages they received and social modeling contribute to their self-efficacy toward ACS (Hypothesis #2); compared with children’s self-efficacy, parents’ self-efficacy on allowing their children to actively commute has stronger correlation with children’s ACS behavior (Hypothesis #3); and there’s a positive correlation between children’s and parents’ self-efficacy (Hypothesis #4).

## Methods

### Study design, participants, and procedures

The current study is part of the Texas Childhood Obesity Prevention Policy Evaluation (T-COPPE) project. The T-COPPE project is a five-year project aimed to evaluate the implementation of two key childhood obesity prevention policies in Texas: 1) the Safe Routes to School (SRTS) program administered through Texas Department of Transportation and 2) federal food allocation package administered through Texas Women, Infants and Children (WIC) Nutrition Program [18]. For evaluation of the SRTS program, researchers used a quasi-experimental design and recruited participants from 79 schools in 28 metropolitan and rural counties across Texas.

Baseline data were collected in 2009, and the post-test data were collected in the 2011–2012 school year. Fourth graders and their parents participated in the project. Student surveys were adapted using available items from other validated surveys and the School Physical Activity and Nutrition (SPAN) surveys [19]. Student assessments included physical activity levels, dietary habits, perceived barriers and self-efficacy to ACS, etc. Parent surveys were adapted using available items from the SRTS parent surveys and other validated measures and included measures of sociodemographics, children’s usual mode of transport to/from school, perceived self-efficacy and barriers to ACS, etc. Both English and Spanish versions of the questionnaires were available depending on participants’ preference. Objective measures, e.g., distance from child’s home to school and land use measures, were captured using geographic Information System (GIS) and the validated T-COPPE school environmental audit tool [20].

For the current study, we utilized the data from the pre-test survey, in which 3315 students and 2055 parents participated. Students whose parents also participated in the survey and provided geocodable home addresses were selected first. To control the effect of long distance as a major barrier to ACS, data of students and parents who lived beyond two miles from school (network distance obtained from GIS) were further excluded. The final analysis included 857 parent/child pairs from 74 schools who lived within two miles of school and didn’t have any disability for walking in urban, suburban, and rural areas. The institutional review boards of The University of Texas and Texas A&M University approved the study.

### Measures

Matching items from parent and child surveys that assessed the same construct(s) were included. Selection of observed variables for each construct was based on their theoretical relevance or the results from reliability and correlation tests [21].

***Children’s self-efficacy*** was a second-order factor collectively measured by three first-order factors: *scheduling self-efficacy, barrier self-efficacy* and *support-seeking self-efficacy*. Items used to measure these factors were adopted from a validated Walking School Bus survey [22].

*Scheduling self-efficacy* was measured by three items asking children how sure they were that they could walk to school to and from school at least once a week, 2–4 days, or every day of the week. The response format included a 3-point Likert scale ranging from “not sure”, “a little sure”, to “very sure.” A reliability analysis for data on these three items resulted in a good Cronbach’s α of 0.83.

*Barrier self-efficacy* was a 6-item subscale asking children about their beliefs in their abilities to walk to school under different difficult situations, e.g., living far, lots of traffic. The items were scaled on a 3-point response format, from “not sure”, “a little sure”, to “very sure.” Cronbach’s α for the six items was 0.84, indicating good internal consistency.

*Support-seeking self-efficacy* was measured by four items, asking children how sure they were that they could walk to school with their parents, with their friends or classmates, by themselves, or without their parents. A reliability test for these items resulted in a Cronbach’s α of 0.73, indicating good internal consistency. Response options included “not sure”, “a little sure”, and “very sure.”

***Children’s previous experience*** of asking for permission to ACS was represented by two items (*ρ* = 0.22, *p* < 0.001), asking children how often they asked their parents if they could walk or ride a bike to school. Responses for the first item included “never”, “sometimes”, “always or almost always” and “I am already walking to school most days.” Responses for the second item included “never”, “sometimes”, “always or almost always”, “I am already riding a bike to school most days” and “I don’t have a bike to ride.” The Spearman’s rank correlation coefficient was reported here rather than Cronbach’s α, which was deemed inappropriate and meaningless for two-item scales [23,24].

***Emotional states*** was measured by two items (*ρ* = 0.53, *p* < 0.001) relating to children’s perceptions about their neighborhood safety (i.e., whether they felt safe walking and biking in the neighborhood during the day). A 4-point response format was used for the two items, ranging from “never” to “all of the time.” The two items were adapted from the validated Amherst Health and Activity Study student survey [25].

***Social persuasion*** was assessed by two items (*ρ* = 0.15, *p* < 0.01). One asked children whether their teachers or other school staff had encouraged them to walk or ride to or from school [26], and the other asked whether schools had a Walking School Bus or a similar program where a group of children walk to or from school together with adults. Response options included “no”, “yes”, and “don’t know.”

***Social modeling*** asked children 1) if many people walked or biked in their neighborhood and 2) how many of their friends usually walked or biked to school (*ρ* = 0.20, *p* < 0.001). Response options for the first items were “never”, “some of the time”, “most of the time”, and “all of the time”. The second item was scaled on a 6-point response format, ranging from “none” to “five or more.” Both of the two items were adapted from previously validated surveys [25,26].

***Environmental constraints*** were represented by seven objectively measured environmental variables (α = 0.67), including home-to-school distance, negative land uses, traffic safety, and social environmental safety en route to school. These variables have been commonly used in active commuting research as indices of environment walkability [27]. Data were derived in 2010–2012 using ArcGIS and ESRI Business Analyst [28].

Distance referred to the shortest network distance from each parent/child pair’s home to school obtained by ArcGIS. The 200 feet buffer along the shortest home-to-school route of each child was used as the spatial unit of measurement for negative land uses and physical and social safety. Negative land uses, obtained from the ESRI Business Analyst, consisted of three composite observed variables, including automobile-related land use, construction and manufacturing-related land use, and general commercial-related land use within home-to-school route buffer. All of the three land use variables were dichotomized as “yes” or “no”, indicating the presence of certain negative land uses or not. It is worth mentioning that in general walkability literature, mixed and commercial-related land use are shown to have positive correlations with walking, especially for utilitarian walking and adult populations. However, such land uses have been shown to play a negative (or inconsistent) role for children (and older adults), because those land uses often come up with additional traffic and other activities that may be perceived unsafe/unattractive for children [29,30]. Therefore, we included commercial-related land use as a measure of negative land uses in this study.

Traffic safety comprised of two items: the presence of highway and the presence of crashes within the route buffer from 2006 to 2009 (0 = No, 1 = Yes), which were obtained from the Texas Department of Transportation. The crash variable was based on a pooled data combining all incidences from year to year, and includes only the collisions involving pedestrians and bicyclists. The presence of sex offenders per acre within the route buffer (0 = No, 1 = Yes) was used as a proxy/indicator of the general social environmental safety, the data of which were derived from the State Department of Public Safety of Texas in 2009. A detailed description of the built environmental variables of the T-COPPE project is available elsewhere [20].

#### Parents’ self-efficacy

In agreement with children’s self-efficacy, parents’ self-efficacy was a second-order factor loaded on three first-order factors: *parents’ scheduling self-efficacy, parents’ barrier self-efficacy* and *parents’ support-seeking self-efficacy.* Matched items for assessing different categories of children’s self-efficacy were used here. Crobach’s α for the three first-order factors were .95, .86, and .76 respectively.

#### ACS

Parents were asked how their 4^th^ grade children arrive at school and leave school on most days of a week, and responses included walk, bike, school bus, family vehicle, carpool, transit, and others. The outcome variable was dichotomized as active or non-active commuter (i.e., whether or not a child walked or biked to or from school on most days of a week).

Control variables included participants’ socioeconomic status (SES), environmental constraints, and school settings. Participants’ SES was measured by three items: number of different types of assistance that a child’s family received, e.g., WIC, Medicaid/Texas Health Steps and food stamps, parental report of the child’s ethnicity (i.e., White or non-White), and car ownership (i.e., whether or not a family had at least one vehicle). School settings included urban/suburban and rural settings.

### Statistical analysis

#### Descriptive statistics

Both parents’ and children’s sociodemographic information were retrieved from parents’ surveys. Prior to conducting more complicated statistical analyses, we examined the frequencies for nominal/ordinal variables and distribution and normality of continuous variables. No statistically significant deviation from the normality assumption was detected in any continuous variable.

#### Modeling

Structural equation modeling (SEM) was selected to test the hypothesized pathways using M*plus* 7.0 [31]. SEM allows researchers to examine relationships among latent variables with multiple observed measures and, more importantly, provides flexibility in testing theory-driven models with empirical data [32]. As a powerful and flexible analytic software, M*plus* handles missing data appropriately and provides estimates for analyzing binary/dichotomous outcome variables, e.g., active or non-active commuter [31]. M*plus* also has the flexibility to estimate mixture modeling (i.e., to simultaneously handle binary, ordinal, and continuous measures). When binary or ordinal variables are present, as in the current study and most health behavioral studies, M*plus* will set up optimal thresholds to ensure a latent factor can have a normal distribution and utilize varying weighted contributions from the variables [33].

Two SEM models were tested for the current study; Model 1 tested Hypotheses #1 and #2, and Model 2 tested Hypotheses #3 and #4. We followed a two-step method for both of the SEM models [33]. In step 1, measurement models were built and evaluated to confirm the factor structure of the latent variables. The mean and variance-adjusted WLS (WLSMV), a more generalized weighted least square based robust estimator, was used for testing measurement models. WLSMV is available in M*plus* and can be applied to a combination of binary, ordered categorical and continuous indicators [31,33]. Higher order CFA modeling was used for children’s self-efficacy and parents’ self-efficacy on both theoretical and empirical bases. Theoretically, Bandura postulated that people’s beliefs in their own abilities are various [12]; empirically, we conducted collinearity diagnostics for observed variables under each construct and found two variables (i.e., “at least once every week” and “every day of the week”) under parents’ self-efficacy had tolerance levels below 0.2 and VIFs greater than 5.0. Given that higher order CFA is a common way to deal with collinearity problems, we introduced higher order factorial structures [34].

In step 2, multilevel modeling was performed to test the hypothesized pathways in the two SEM models. A two-level structure of children nested within schools was employed based on the assumption that similar active commuting patterns may be clustered among children attending the same schools [35]. Again, WLSMV was used as the recommended and default estimator in M*plus* for modeling binary outcomes. Model fit was evaluated based on the following fit indices: the Bentler comparative fit index (CFI), the Tucker-Lewis index (TLI), the root mean square error of approximation (RMSEA) and its 90% confidence interval, and the weighted root mean square residual (WRMR) [31,36]. To improve model fit, we re-specified the models based on modification indices. Item-to-factor loadings, factor correlations, and path coefficients for the measurement and structural models were inspected for sign and/or for magnitude.

#### Missing data

No missing value is present for objective data obtained by GIS, including distance, environmental constraints, and school setting. For the other observed variables, missing data ranged from 0% to 6.0%. By default, data containing missing values are listwise deleted when modeling binary outcome using WLSMV estimator in M*plus* [31].

## Results

### Sample characteristics and descriptive statistics

Sample characteristics are presented in Table 1. Of the 857 4th grade students, 49.2% were boys and 50.3% were girls; and the majority were non-White (79.9%). Approximately 70% of the children’s families received at least one type of assistance. Over 80% of the children were from schools located in urban or suburban areas, with only 13.9% from rural schools. Over 18% of the students were active commuters, while 78.8% were not. Most families (92.5%) owned at least one vehicle; only 3.9% had no vehicle at home.

**Table 1:** **Sociodemographic characteristics of participants**

**Characteristics**	**% or mean (SD)**
**Child’s gender**	
Boy	49.2
Girl	50.3
**Child’s ethnicity**	
White	19.5
Non-white	79.9
**Number of assistance a family received**	1.67 (1.49)
**Car ownership**	
At least one vehicle	92.5
No vehicle	3.9
**School settings**	
Urban/suburban	86.1
Rural	13.9
**Modes of commuting to school**	
Active (i.e., walk or bike)	18.1
Non-active	78.8

Table 2 presents the coding scheme and descriptive statistics for latent and observed variables that were used. Most of the observed variables were categorical or ordinal, and few were continuous variables.

**Table 2:** **Coding scheme and descriptive statistics for latent and observed variables (N = 857)**

**Description**	**Latent and observed variables**	**Coding schemes and descriptive statistics**
**Types of children’s self-efficacy**	*I’m sure that I can walk to or from school:*	
Scheduling self-efficacy	At least once every week	0: Not sure (48.8%), 1: A little sure (21.7%), 2: Very sure (26.1%)
	At least 2–4 days of the week	0: Not sure (54.7%), 1: A little sure (19.1%), 2: Very sure (23.1%)
	Every day of the week	0: Not sure (57.9%), 1: A little sure (13.0%), 2: Very sure (24.9%)
Barrier self-efficacy	Even if I live far from school	0: Not sure (69.3%), 1: A little sure (15.2%), 2: Very sure (13.4%)
	Even if there is a lot of traffic	0: Not sure (70.6%), 1: A little sure (16.3%), 2: Very sure (10.3%)
	Even if it is hot outside	0: Not sure (43.2%), 1: A little sure (25.2%), 2: Very sure (28.8%)
	Even if it is cold outside	0: Not sure (56.4%), 1: A little sure (22.4%), 2: Very sure (18.7%)
	Even if it is raining outside	0: Not sure (68.1%), 1: A little sure (15.2%), 2: Very sure (13.7%)
	Even if my friends or classmates do not walk to school	0: Not sure (49.9%), 1: A little sure (20.3%), 2: Very sure (26.4%)
Support-seeking self-efficacy	With my parents	0: Not sure (37.3%), 1: A little sure (19.5%), 2: Very sure (40.1%)
	With my friends or classmates	0: Not sure (39.3%), 1: A little sure (19.5%), 2: Very sure (38.3%)
	By myself	0: Not sure (57.1%), 1: A little sure (16.2%), 2: Very sure (25.1%)
	Without my parents	0: Not sure (52.7%), 1: A little sure (16.7%), 2: Very sure (27.2%)
**Sources of children’s self-efficacy**		
Previous experience of asking for permission to ACS	How often do you ask your parents if you can walk to school?	0: Never (50.1%), 1: Sometimes (22.5%), 2: Always (11.4%); 3: Already walked to school (14.8%)
	How often do you ask your parents if you can bike to school?	0: I do not have a bike (19.7%), 1: Never (49.5%), 2: Sometimes (16.3%), 3: Always (9.8%), 4: Already biked to school (4.1%)
Emotional states	Do you feel safe walking in your neighborhood during the day?	0: Never (15.5%), 1: Sometimes (23.8%), 2: Most of the time (20.9%); 3: All of the time (39.1%)
	Do you feel safe riding a bike in your neighborhood during the day?	0: Never (15.5%), 1: Sometimes (20.4%), 2: Most of the time (18.8%); 3: All of the time (44.8%)
Social persuasion	Have your teachers or other school staff encouraged you to walk or ride to or from school?	0: No (67.2%), 1: Yes (13.3%), 2: Don’t know (18.6%)
	Does your school have a Walking School Bus or a similar program?	0: No (41.9%), 1: Yes (15.3%), 2: Don’t know (42.2%)
Social modeling	Do many people walk or ride bikes in your neighborhood?	0: Never (7.1%), 1: Sometimes (46.8%), 2: Most of the time (25.1%); 3: All of the time (20.8%)
	How many of your friends usually walk or ride a bike to school?	Mean: 1.77, SD:1.82
**Environmental constraints**	Percentage of highway	0: No (82.4%), 1: Yes (17.6%)
	Automobile related land use	0: No (66.7%), 1: Yes (33.3%)
	Construction and manufacturing related land use	0: No (64.9%), 1: Yes (35.1%)
	General commercial related land use	0: No (58.0%), 1: Yes (42.0%)
	Presence of crashes per acre	0: No (67.9%), 1: Yes (32.1%)
	Presence of sex offenders per acre	0: No (72.1%), 1: Yes (27.9%)
	Network distance	Mean: .80, SD: .48
**Types of parents’ self-efficacy**	*I’m sure that I can allow my child to walk to or from school:*	
Parent scheduling self- efficacy	At least once every week	0: Not sure (59.7%), 1: A little sure (16.5%), 2: Very sure (18.1%)
	At least 2–4 days of the week	0: Not sure (64.6%), 1: A little sure (13.8%), 2: Very sure (15.5%)
	Every day of the week	0: Not sure (70.1%), 1: A little sure (10.5%), 2: Very sure (13.7%)
Parent barrier self-efficacy	Even if we live far from school	0: Not sure (87.8%), 1: A little sure (4.9%), 2: Very sure (2.9%)
	Even if there is a lot of traffic	0: Not sure (86.1%), 1: A little sure (6.3%), 2: Very sure (2.8%)
	Even if it is hot outside	0: Not sure (63.5%), 1: A little sure (20.7%), 2: Very sure (11.1%)
	Even if it is cold outside	0: Not sure (72.0%), 1: A little sure (16.9%), 2: Very sure (6.1%)
	Even if it is raining outside	0: Not sure (83.8%), 1: A little sure (6.7%), 2: Very sure (3.5%)
	Even if other children do not walk to school	0: Not sure (75.1%), 1: A little sure (12.6%), 2: Very sure (6.9%)
Parent support-seeking self-efficacy	With me	0: Not sure (27.5%), 1: A little sure (17.2%), 2: Very sure (50.6%)
	With friends or classmates	0: Not sure (55.8%), 1: A little sure (20.4%), 2: Very sure (18.6%)
	Alone, without other children or adults	0: Not sure (78.4%), 1: A little sure (8.1%), 2: Very sure (7.9%)
	Without me	0: Not sure (67.8%), 1: A little sure (14.9%), 2: Very sure (11.6%)

### Measurement and structural models

Measurement models were assessed with CFA to confirm the factor structures of all model constructs. Standardized item-to-factor loadings were examined and variables that had poor factor loadings (below 0.30) and non-significant relationships (*p* > 0.05) with individual latent factor were removed [37].

#### Structural model 1 for children’s self-efficacy

Two hypotheses were tested in structural model 1: children’s self-efficacy is positively associated with their ACS (Hypothesis #1), and children’s previous experience of asking for permission to ACS, emotional states, the persuasive messages they received, and social modeling contribute to their self-efficacy toward ACS (Hypothesis #2).

Table 3 displays the standardized item-to-factor correlations for Structural Model 1, with weak relationships removed. The latent factor, *previous experience of asking for permission to ACS*, was removed from further modeling analyses because of the poor factor loadings of the two items attempting to refer it. Presence of sex offenders within route buffer per acre was further removed because of small factor loading. In order to improve model fit, we created another latent factor, social economic disadvantage, which was captured by the number of assistances that a child’s family received and child’s ethnicity. Car ownership was deleted as a measure of social economic disadvantage in the measurement model and as a control variable in the SEM model because of its unbalanced distribution (only 3.9% of families did not have a vehicle), which might cause the models to be misspecified [31,33].

**Table 3:** **Standardized item-to-factor correlations for structural model 1: children’s self-efficacy model (N = 857)**

**Description**	**Latent factor/Observed variables**	**Factor loading**	**P-value**
**Types of children’s self-efficacy**	**Scheduling Self-efficacy (3 items)**		
	*I’m sure that I can walk to and from school:*		
	At least once every week	.78 (.02)	.000
	At least 2–4 days of the week	.87 (.02)	.000
	Every day of the week	.91 (.02)	.000
	**Barrier Self-efficacy (6 items)**		
	Even if I live far from school	.69 (.03)	.000
	Even if there is a lot of traffic	.70 (.03)	.000
	Even if it is hot outside	.83 (.02)	.000
	Even if it is cold outside	.80 (.02)	.000
	Even if it is raining outside	.77 (.03)	.000
	Even if my friends or classmates do not walk to school	.87 (.02)	.000
	**Support-seeking Self-efficacy (4 items)**		
	With my parents	.40 (.05)	.000
	With my friends or classmates	.80 (.02)	.000
	By myself	.91 (.01)	.000
	Without my parents	.91 (.01)	.000
**Sources of children’s self-efficacy**	**Emotional States (2 items)**		
	Do you feel safe walking in your neighborhood during the day?	.83 (.05)	.000
	Do you feel safe riding a bike in your neighborhood during the day?	.64 (.05)	.000
	**Social Persuasion (2 items)**		
	Have your teachers or other school staff encouraged you to walk or ride to or from school?	.78 (.26)	.002
	Does your school have a Walking School Bus or a similar program?	.38 (.12)	.003
	**Social Modeling (2 items)**		
	Do many people walk or ride bikes in your neighborhood?	.44 (.06)	.000
	How many of your friends usually walk or ride a bike to school?	.46 (.07)	.000
**Social economic disadvantage**	Number of assistance that a child’s family received	.47 (.09)	.000
	Ethnicity (White or non-white)	.61 (.12)	.000
**Environmental constraints**	Percentage of highway (binary)	.64 (.09)	.000
	Auto-related land use (binary)	.73 (.08)	.000
	Construction and manufacturing land use (binary)	.46 (.07)	.000
	General commercial land use (binary)	.68 (.07)	.000
	Presence of crashes per acre (binary)	.31 (.08)	.001
	Network distance	.87 (.07)	.000

**Figure 2:**
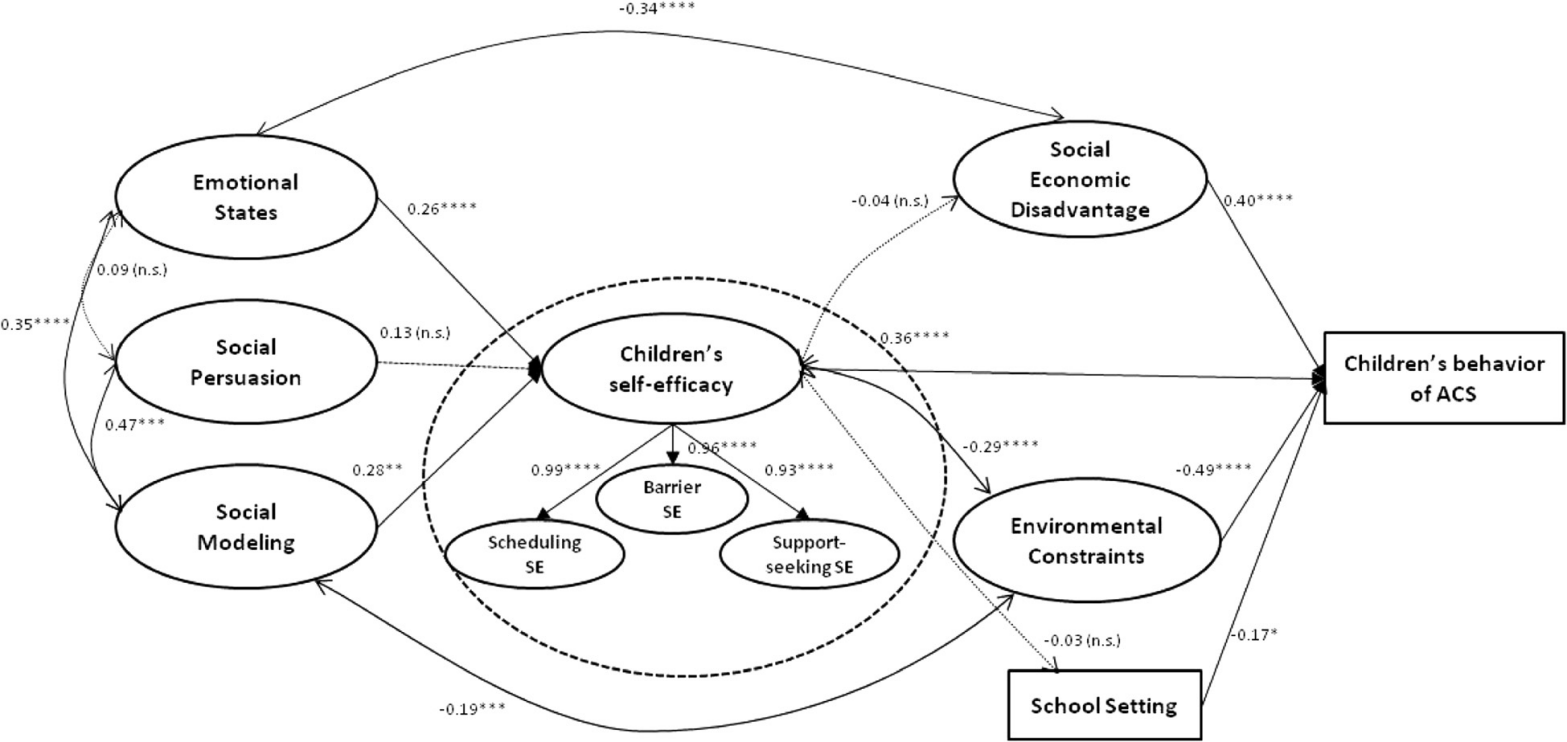
**Structural model 1 for children’s self-efficacy (N = 857).** Note: Parameter estimates are standardized regression weights. A regression weight with a positive sign means the expected value of the dependent variable (i.e., child behavior of ACS) is increased when the predictor value increases. Model Fit Statistics: CFI = 0.99; TLI = 0.99; RMSEA = 0.02; WRMR = .84. *p ≤ 0.05, **p ≤ 0.01, ***p ≤ 0.005, ****p ≤ 0.001, n.s. = not significant.

Figure 2 displays the final structural model, which proved excellent fit to the data (CFI = 0.99, TLI = 0.99, RMSEA = 0.02, WRMR = 0.84). Among this sample of children, the model accounted for 65.4% of the variance in the final outcome (i.e., ACS). As hypothesized, the relationship between children’s self-efficacy and their ACS behavior was significant and positive (β = 0.26, p < 0.001). Emotional states (β = 0.36, p < 0.001) and social modeling (β = 0.28, p < 0.01) had direct pathways to children’s self-efficacy, but there was no direct pathway between social persuasion and children’s self-efficacy (β = 0.13, p = 0.25). Moreover, emotional states (β = 0.09, p = 0.001) and social modeling (β = 0.10, p = 0.028) also had significant indirect effects on children’s active commuting behavior via children’s self-efficacy. In other words, the effects of emotional states and social modeling on children’s ACS were mediated by children’s self-efficacy.

All of the three latent and observed control variables, i.e., social economic disadvantage (β = 0.40, p < 0.001), environmental constraints (β = −0.49, p < 0.001), and school setting (β = −0.17, p = 0.029), had statistically significant direct effects on children’s ACS. Specifically, children from social economic disadvantaged families were more likely to walk or bike to school compared with those from higher social economic families. Environmental constraints were negatively associated with children’s ACS; children with fewer environmental constraints were more likely to walk or bike to school. Compared with children from urban or suburban schools, children from rural schools were more likely to commute actively. The relationship between environmental constraints and children’s self-efficacy was also significant (β = −0.29, p < 0.001), indicating that children’s self-efficacy increased when environmental constraints decreased.

Other significant relationships included social economic disadvantage and emotional states (β = −0.34, p < 0.001), social modeling and emotional states (β = 0.35, p < 0.001), social persuasion and social modeling (β = 0.47, p = 0.004), and school setting and social modeling (β = −0.19, p < 0.001).

#### Structural model 2 for children’s self-efficacy vs. parents’ self-efficacy

The other two hypotheses were tested in structural model 2: compared with children’s self-efficacy, parents’ self-efficacy on allowing their children to actively commute has a stronger correlation with children’s ACS behavior (Hypothesis #3), and there’s a positive correlation between children’s and parents’ self-efficacy (Hypothesis #4).

Table 4 exhibits the standardized item-to-factor correlations for Structural Model 2, with two observed variable with low factor loadings removed (“I’m sure that I can walk to or from school even if it is raining outside” and “I’m sure that I can allow my child to walk to or from school even if it is raining outside”). Although the item “I’m sure I can walk or bike to or from school with my parents” had a factor loading less than 0.3, it was statistically significant (p < 0.001). Further considering its theoretical importance further, we decided to retain this item in the model.

**Table 4:** **Standardized item-to-factor correlations for structural model 2: children’s self-efficacy** ***vs.*** **parents’ self-efficacy model (N = 857)**

**Description**	**Latent factor/Observed variables**	**Factor loading**	**P-value**
**Types of children’s self-efficacy**	**Scheduling Self-efficacy (3 items)**		
	*I’m sure that I can walk to and from school:*		
	At least once every week	.77 (.02)	.000
	At least 2–4 days of the week	.87 (.02)	.000
	Every day of the week	.92 (.01)	.000
	**Barrier Self-efficacy (6 items)**		
	Even if I live far from school	.68 (.03)	.000
	Even if there is a lot of traffic	.69 (.03)	.000
	Even if it is hot outside	.82 (.02)	.000
	Even if it is cold outside	.78 (.032)	.000
	Even if my friends or classmates do not walk to school	.87 (.02)	.000
	**Support-seeking Self-efficacy (4 items)**		
	With my parents	.28 (.05)	.000
	With my friends or classmates	.77 (.03)	.000
	By myself	.87 (.02)	.000
	Without my parents	.88 (.02)	.000
**Types of parents’ self-efficacy**	**Scheduling Self-efficacy (3 items)**		
	*I’m sure that I can allow my child to walk to or from school*		
	At least once every week	.96(.01)	.000
	At least 2–4 days of the week	.98 (.01)	.000
	Every day of the week	.98 (.01)	.000
	**Barrier Self-efficacy (6 items)**		
	Even if we live far from school	.67 (.03)	.000
	Even if there is a lot of traffic	.76 (.03)	.000
	Even if it is hot outside	.88 (.02)	.000
	Even if it is cold outside	.82 (.02)	.000
	Even if other children do not walk to school	.93 (.02)	.000
	**Support-seeking Self-efficacy (4 items)**		
	With me	.54 (.04)	.000
	With friends or classmates	.90 (.01)	.000
	Alone, without other children or adults	.90 (.02)	.000
	Without me	.92 (.01)	.000
**Social economic disadvantage**	Number of assistance that a child’s family received	.36 (.12)	.003
	Ethnicity (White or non-white)	.82 (.25)	.001
**Environmental constraints**	Percentage of highway (binary)	.64 (.08)	.000
	Auto-related land use (binary)	.70 (.08)	.000
	Construction and manufacturing land use (binary)	.49 (.07)	.000
	General commercial land use (binary)	.65 (.07)	.000
	Presence of crashes per acre (binary)	.31 (.08)	.001
	Network distance	.90 (.05)	.000

**Figure 3:**
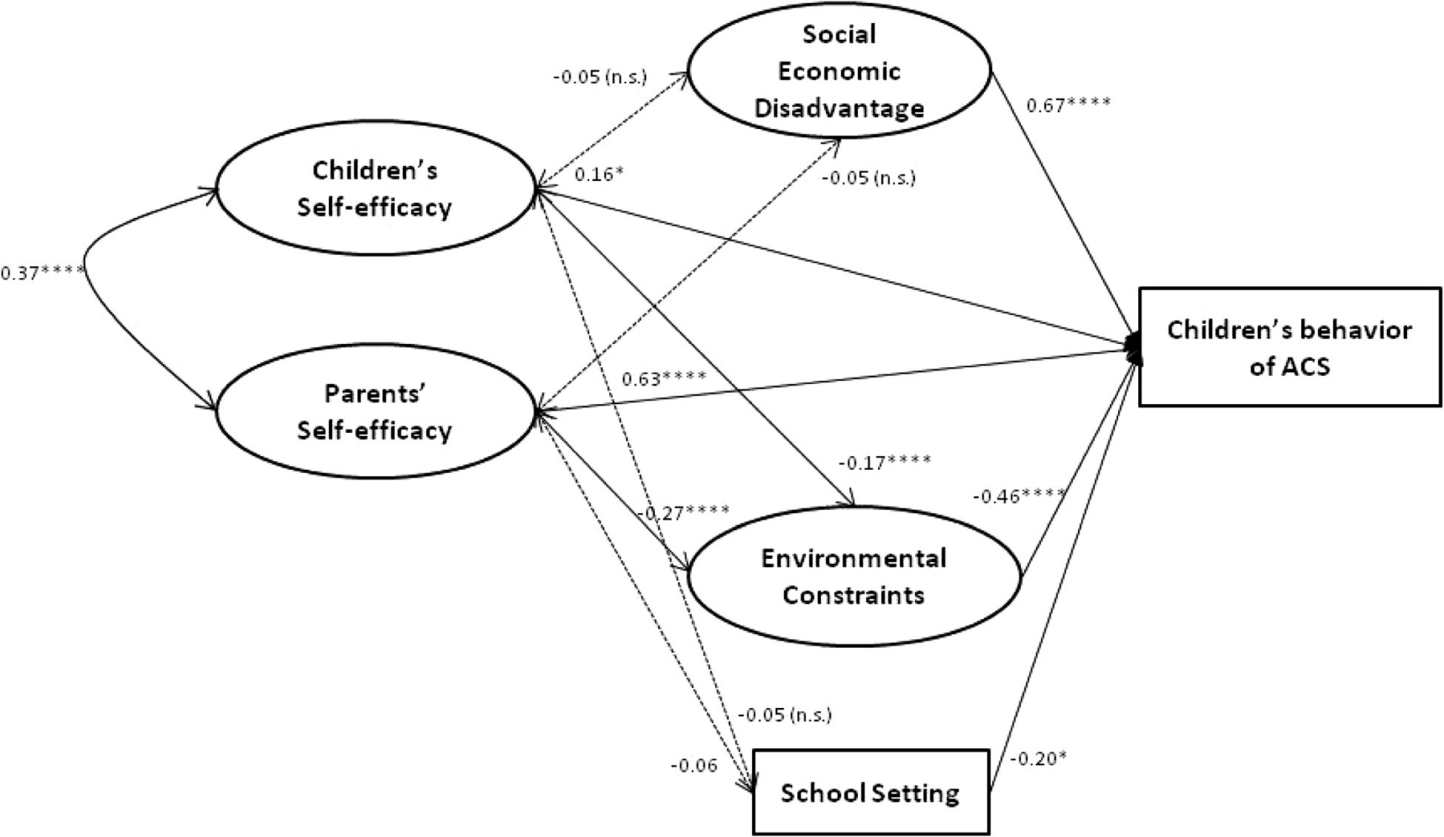
**Structural model 2 for children’s self-efficacy** ***vs.*** **parents’ self-efficacy (N = 857).** Note: Parameter estimates are standardized regression weights. A regression weight with a positive sign means the expected value of the dependent variable (i.e., child behavior of ACS) is increased when the predictor value increases. Model Fit Statistics: CFI = 0.995; TLI = 0.995; RMSEA = 0.02; WRMR = .98. *p ≤ 0.05, **p ≤ 0.01, ***p ≤ 0.005, ****p ≤ 0.001, n.s. = not significant.

Figure 3 depicts the final structural model, which demonstrated good fit to the data (CFI = 0.995, TLI = 0.995, RMSEA = 0.02, WRMR = 0.98). Overall, the model accounted for 82.2% of the variance in the final outcome variable ACS. As we hypothesized, compared with children’s self-efficacy (β = 0.16, p < 0.001), parents’ self-efficacy (β = 0.63, p < 0.001) had a stronger influence on children’s active commuting behavior. There was also a significant correlation between children’s self-efficacy and parents’ self-efficacy (β = 0.37, p < 0.001). In agreement with Structural Model 1, all of the three control variables, i.e., social economic disadvantage (β = 0.67, p < 0.001), environmental constraints (β = −0.46, p < 0.001), and school setting (β = −0.20, p < 0.001), had statistically significant direct effects on children’s self-efficacy. The directions of the relationships between the control variables and ACS were the same with those in Structural Model 1.

Other significant relationships included environmental constraints and children’s self-efficacy (β = −0.17, p < 0.001), and environmental constraints and parents’ self-efficacy (β = −0.27, p < 0.001).

## Discussion

This study is one of the first to simultaneously model the relationships between children’s self-efficacy, parents’ self-efficacy, social economic disadvantage, environmental constraints, and children’s ACS.

Our study confirmed the determinant roles of both the children’s and parents’ self-efficacy in children’s active commuting behavior and verified that, compared with children’s self-efficacy, parents’ self-efficacy had a greater effect on children’s active commuting behavior. The models also revealed multiple personal, social, and environmental factors that can influence both children’s self-efficacy and children’s ACS behavior.

In agreement with previous investigations showing that school age children’s perceived self-efficacy is related to their physical activity [11,38], we found that children’s beliefs in their own abilities to overcome various barriers directly predicted their active commuting behavior. Quite often, children’s perceptions and attitudes as “key informants” in matters related to their health are ignored, based on the assumption that children are not mature enough to self-report their views [39,40]. Subsequently, the prevailing approach to researching children’s experience is grounded in “research on” rather than “research with” children [39,40]. The positive association that we revealed between children’s self-efficacy and ACS may reassure health behavior researchers that children had the cognitive abilities to contribute meaningful and insightful research data. We propose, therefore, that more sophisticated child-centered ACS studies be conducted to assess self-reported psychological variables with children. Further, future interventions targeted at promoting ACS also need to include strategies that can increase children’s self-efficacy.

The findings of our study proposed four potential strategies that can be applied to increase children’s self-efficacy. First, community-based interventions are encouraged to secure neighborhood safety, which promises to develop children’s self-efficacy. As reported in our study, when children felt safe walking or biking in their neighborhood, they were more confident in themselves and thereby more likely to be active commuters. We recommend that schools, families, and communities work collaboratively to develop effective monitoring mechanisms to foster a sense of security in children.

Second, children’s self-efficacy may be promoted by increased exposure to supportive role models and positive peer influence, as substantiated by the positive effect between social modeling and children’s self-efficacy. Programs should attempt to involve adults, particularly parents, as role models for children through active commuting. An example of such a program is the Walking School Bus program, in which a group of students walking to/from school with adults [41]. By engaging parents and children in active commuting together, the Walking School Bus program may provide enough social motivation to increase children’s desire and self-efficacy to actively commute [41].

Despite the potential importance of the Walking School Bus program, it is worth mentioning that social persuasion, measured by school encouragement and Walking School Bus program availability at schools, was not a significant predictor of children’s self-efficacy in this study. However, the small number of students (15.3%) reporting that their schools had such a program might have limited statistical power to detect any difference that might exist. Further considering that 84.1% of the students mentioned either their schools did not have such a program or they didn’t know whether there’s such an initiative in their schools, we recommend that schools raise awareness and increase the practice of the program among students.

Third, the positive correlation between children’s self-efficacy and parents’ self-efficacy implied that children’s self-efficacy can be promoted by increasing parents’ self-efficacy. Limited by the use of secondary data, we didn’t investigate the sources of parents’ self-efficacy. We call for future studies to examine factors that can influence parents’ self-efficacy to facilitate effective interventions for promoting children’s self-efficacy and subsequently active commuting behavior.

Fourth, children’s self-efficacy can be strengthened by reducing physical and social environmental constraints. Previous research has established the effects of the environmental factors included in our study on children’s active commuting behavior, but no study has examined the relationship between these factors and children’s self-efficacy toward ACS [27,42]. The negative association between environmental constraints and children’ self-efficacy suggests a need for approaches to improve physical and social environments. For example, land use plans need to be strategized to allow for easy walking or biking in school areas; traffic safety should be improved to reduce the number of crashes; and parents are encouraged to send their children to nearby schools to facilitate active commuting.

In agreement with findings from previous studies, this study showed a positive association between parents’ self-efficacy and children’s ACS [5,14]. And, not surprisingly, compared with children’s self-efficacy, parents’ self-efficacy played a more important role in determining children’s active commuting behavior. This supported the previous hypothesis that parents are usually the main decision-makers for their children’s commuting mode choice to school [43]. Nevertheless, children’s self-efficacy can have a potential influence on their parents’ self-efficacy, as established by the significant association between the children’s and parents’ self-efficacy. Therefore, we emphasize that children’s perceived self-efficacy be considered when planning interventions for ACS.

Congruent with previous research, there is a significant association between participants’ social economic disadvantage and children’s active commuting behavior in this study. Compared with White children and children from a high SES background, non-White children and children from social economic disadvantaged families were more likely to be active commuters [44]. Considering that children from social economic disadvantaged families were less likely to feel safe walking or biking in their neighborhoods, as reported in this study, we call for future ACS interventions targeted at improving safety in low SES neighborhoods in order to promote ACS.

Previous studies have reported that children living in urban neighborhoods with supportive infrastructure (e.g., availability of sidewalks and positive land uses) and social norms were more likely to walk or bike to schools [44]. However, our data suggested that children from rural schools were more likely to be active commuters. With a small percentage of children from rural schools (13.9%), we failed to conduct a multiple group comparison; future studies with larger sample sizes are needed to detect the underlying reasons preventing rural children from walking or biking to school.

### Limitations and strengths

The findings of this study should be interpreted in light of the following limitations. First, this is a secondary analysis of data from a larger study, thus we had no control of variables. For example, we had several latent constructs assessed with only two items, which might not have enough power to capture the multidimensional nature of the construct. The validity of the constructs could be improved by measuring a more comprehensive list of variables. Second, all the variables that we used to measure self-efficacy were ordinal. This was inconsistent with Bandura’s guidelines that measurement should capture the strength of self-efficacy [45], which is usually measured on a scale ranging from 0% to 100%. However, refinement of a psychometric survey is typical in social and behavioral sciences, and a set of ordinally scaled items is often used to assess a psychological construct [46]. Third, we didn’t compare the relationships between different types of self-efficacy (i.e., scheduling self-efficacy, barriers self-efficacy and support-seeking SE), and children’s ACS, as it’s not part of our research questions. Future studies are needed to investigate and compare the relationships among different types of self-efficacy and their influences on children’s ACS. Fourth, some environmental variables investigated in this study (e.g., crashes, the presence of sex offenders) were based on pooled data, which may not be sophisticated enough. Future studies are warranted to include more detailed variables such the presence of footpaths, bike tracks, and traffic-calming features, as well as specific crime incidences as measures of traffic safety and social environmental safety.

Nevertheless, this study has several major strengths. First, it was built upon well-established social cognitive framework, which guided the data analysis and interpretation. Second, we used SEM for data analysis, which allows for simultaneous assessment of relationships among different factors and provides flexibility in testing theory-driven models. Third, we included both children and parents as participants, which allowed for direct comparisons. Fourth, we included both perceived and objective measures in the study, which provided a more comprehensive context for examining predictors of children’s ACS.

## Conclusions

Findings of this study confirmed the predictive ability of children’s self-efficacy on their active commuting behavior and suggested potential interventions that may be effective in promoting children’s self-efficacy. While we supported the role of parents as the key decision-makers regarding ACS, this study demonstrated that children can also contribute valuable research data and their beliefs in their own capabilities should be considered when planning ACS programs. The work reported here provides support for the continuing exploration of the role of self-efficacy in children’s ACS.
